# Automatic titration detection method of organic matter content based on machine vision

**DOI:** 10.1098/rsos.250234

**Published:** 2025-07-02

**Authors:** Bingjie Zhang, Meng Li, Qing Song, Lujian Xu

**Affiliations:** ^1^ University of Jinan, Jinan, People’s Republic of China

**Keywords:** organic matter detection, machine learning, endpoint detection, supervised classification

## Abstract

This article proposes an automatic titration algorithm for organic matter content detection based on machine vision, which addresses the disadvantages of high risk factor, strong odour, significant pollution to laboratory environment and slow efficiency of manual titration in organic matter detection. First, by analysing the colour change characteristics during the titration process, machine learning techniques are used to classify the titration speed, and a titration experiment state recognition model is constructed to divide the titration speed into four categories and improve titration efficiency; Second, through a large number of titration experiments to collect relevant data and extract key feature parameters, an efficient titration algorithm based on histogram similarity was designed to accurately identify titration endpoints and improve detection accuracy. This study not only solves the limitations of manual operation in traditional titration methods, but also provides new ideas and methods for the automation and intelligence of chemical titration. The test results showed that the device had a titration error of less than 0.2 ml and was more efficient than manual titration. When comparing the results with manual titration, no statistically significant difference was observed when paired *t*-test was applied at a 95% confidence level. Therefore, it has been confirmed that it has good recognition rate and control accuracy.

## 1. Introduction

As a traditional industry dominated by manual testing, chemistry has gradually adopted automated intelligent testing methods with the development of automation technology. Chemical titration is a commonly used quantitative analysis method used to determine the concentration of a specific chemical substance in a solution. It is a simple, rapid and widely used quantitative analysis method and an important means of chemical analysis [1]. At present, organic matter detection is of vital importance in agriculture, environmental protection and scientific research. It is responsible for accurately determining the organic matter content in samples such as soil, water and plants. Organic matter can improve soil structure and soil fertility. The determination of organic matter content mainly adopts the potassium dichromate oxidation-volume method, which is a visual titration method [2]. Although this method is relatively simple in operation and has less interference, it also has some significant limitations:

(1) The safety and environmental pollution issues of manual operation: potassium dichromate sulfuric acid solution has a high level of danger, and its strong irritating odour not only pollutes the laboratory environment, but also poses a potential threat to the health of operators.
(2) The manual titration process is tedious and time-consuming, and the accuracy and consistency of the results are difficult to guarantee: the operator needs to manually control the droplet acceleration of the titrant and closely observe the change in solution colour with the naked eye at all times to accurately determine the titration endpoint. This process is highly susceptible to various factors such as the operator’s personal experience and laboratory environment, making it difficult to effectively guarantee the accuracy and consistency of titration results.
(3) Difficulty in determining the endpoint of titration. In some complex titration experiments, incomplete local reactions may occur. This can cause the colour of the solution to return to its original state after stirring, resulting in problems such as untimely or inaccurate determination of the titration endpoint. This phenomenon not only increases the delay of the experiment, but also makes the observation of colour changes and endpoint judgement during the titration process more complicated, further increasing the difficulty of the experiment.

Therefore, it is particularly important to accurately identify the solution automatically, realize automatic dripping without human intervention and improve the accuracy and efficiency of the titration [3].

In order to solve the above problems, the introduction of automation technology provides a new possibility for chemical titration. Over the past half century, the research on automatic titration technology has made significant progress. The potentiometric method [4,5], conductivity method [6], coulometric method [7], spectroscopy [8], temperature method [9], digital image-based measurement [10–12] and other methods have been widely used in titration analysis as analytical response techniques. These methods successfully solve the difficulties in visual recognition of traditional titration endpoints by monitoring changes in the potential, conductivity, current, spectral characteristics, temperature or visual characteristics of the solution and significantly improve the accuracy and precision of titration analysis. For example, Thajee *et al.* [13] proposed an electrochemical-colorimetric dual detection system; Lima *et al.* [14] proposed to determine the titration endpoint by calculating the Pearson correlation coefficient between the H value of the initial frame and each subsequent frame; Kholeif [15] proposed a differential method to determine the endpoint by the potentiometric titration curve; Hu *et al.* [16] proposed an automatic titration system for batch measurement of total alkalinity; Nazarenko [17] applied optical sensors to titration experiments; Xueting *et al.* [18] compared the area values of the inner circle and the halo ring according to the difference in blue absorption when detecting the effective content of bentonite in old sand clay, and compared the obtained area ratio with the set standard value to determine whether the titration endpoint has been reached; Siqueira *et al.* [19] used a precise automatic titration programme with low sharpness and dichroism to detect the titration endpoint. Berasarte *et al.* [20] used digital colourimetry and a paper-based analytical device for acetic acid microtitration; Ruttanakorn *et al.* [21] first developed a smartphone-based linear piecewise curve technique of RGB versus titrant volume.

Current research has made progress in improving titration efficiency and accuracy [22–25], but most of it still focuses on potentiometric titration [26–29] or colour sensors [30–32]. However, for the organic matter content detection experiment in this article, both methods are not applicable for the following reasons. The potassium dichromate volumetric method involves strong acidic media (such as concentrated sulfuric acid) and high-temperature digestion processes, with a large number of oxidized ions (such as Cr³^+^, Fe³^+^) and reduced substances in the reaction system. These ions may contaminate the electrode surface, causing potential signal drift or shortened electrode lifespan, interfering with the endpoint determination of potentiometric titration, while colour sensors are limited by gradient colour and dynamic titration requirements. In contrast, machine vision-based methods have shown potential in various industries [33]. For example, [10–12] combine different machine vision methods and use cameras instead of human eyes for titration in chemical experiments. However, current research still lacks systematic classification and regulation of titration speed, and in the future, machine vision can be used to dynamically adjust droplet acceleration.

Based on this, this article proposes an automatic titration algorithm for organic matter content detection based on machine vision, aiming to solve many problems in traditional titration methods through automation technology, especially the limitations of manual operation and the accuracy of titration endpoint judgement. The main objectives of the research include: (i) By analysing the characteristics of the colour value curve, using machine learning to classify the titration speed, establishing a titration experiment state recognition model and improving the titration efficiency; (ii) Through a large number of titration experiments, relevant experimental data are collected, important characteristic parameters are extracted and an efficient titration algorithm is designed to achieve the function of accurately finding the chemical titration endpoint and improve the detection accuracy.

Specifically, this article will use machine learning technology to process and analyse the data of solution colour changes, automatically identify the different stages of the titration process and accurately determine the titration endpoint without human intervention. This method can not only effectively reduce human errors in experiments, reduce experimental risks and environmental pollution, but also greatly improve the efficiency of titration experiments and the accuracy of test results. At the same time, the research results of this article will provide new ideas and methods for the automation and intelligent development of chemical analysis, and promote the modernization of traditional chemical analysis technology.

## 2. Material and methods

### 2.1. Data collection

This article takes the measurement of organic matter content in organic fertilizer as an example [34]. Stir the fertilizer evenly, use an instrument to collect samples at different points and layers and after mixing evenly, send it to the sample processing room for processing. Then, air-dry and crush the tested organic fertilizer samples, and then use the potassium dichromate oxidation-volume method [35]. The principle of this method is to evenly mix a certain amount of potassium dichromate solution and concentrated sulfuric acid, heat them, oxidize the organic carbon in the organic fertilizer and titrate the remaining potassium dichromate solution with ferrous sulfate solution, use o-phenanthroline as an indicator, and use silicon dioxide as an additive to perform a blank test. The organic carbon content is calculated based on the amount of potassium dichromate consumed, and multiplied by 1.724 to get the organic matter content. The chemical reactions during oxidation and titration are as follows:


(2.1) $$2{\text{K}}_{2}\text{C}{\text{r}}_{2}{\text{O}}_{7}+3\text{C}+8{\text{H}}_{2}\text{S}{\text{O}}_{4}=2{\text{K}}_{2}\text{S}{\text{O}}_{4}+2\text{C}{\text{r}}_{2}{\left(\text{S}{\text{O}}_{4}\right)}_{3}+3\text{C}{\text{O}}_{2}↑+8{\text{H}}_{2}\text{O},$$

(2.2) $${\text{K}}_{2}\text{C}{\text{r}}_{2}{\text{O}}_{7}+6\text{F}\text{e}\text{S}{\text{O}}_{4}+7{\text{H}}_{2}\text{S}{\text{O}}_{4}={\text{K}}_{2}\text{S}{\text{O}}_{4}+\text{C}{\text{r}}_{2}{\left(\text{S}{\text{O}}_{4}\right)}_{3}+3\text{F}{\text{e}}_{2}{\left(\text{S}{\text{O}}_{4}\right)}_{3}+7{\text{H}}_{2}\text{O}.$$

The reagents that need to be prepared include: c (1/6K _2_Cr_2_O_7_) = 0.8 mol l^−1^ standard solution, c (FeSO_4_) = 0.2 mol l^−1^ sulfuric acid standard solution, o-phenanthroline indicator and concentrated sulfuric acid.

**Figure 1.**
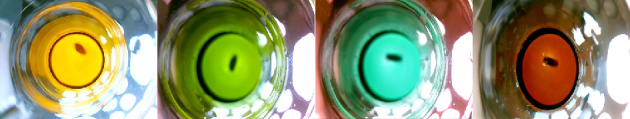
Data collection process.

Weigh a certain amount of air-dried organic fertilizer (organic carbon content is not more than 15 mg), place it in a 500 ml conical flask, accurately add 0.8–1.0 mol l^−1^ potassium dichromate sulfuric acid solution (because concentrated sulfuric acid is highly corrosive, you need to pay attention to safety when using concentrated sulfuric acid), add a bent neck funnel, place it in a boiling water bath and heat for 30 min, take it out and let it cool to room temperature, transfer it to a 250 ml volumetric flask without damage, make up the volume, measure 50 ml of the mixed solution in the conical flask and add water to 100 ml. Add 8–10 drops of o-phenanthroline indicator to it and titrate with ferrous sulfate standard solution. During the titration process, since potassium dichromate is orange, the colour of the solution is mainly orange at the beginning. As the titration proceeds, the solution gradually shows green. When it is close to the endpoint, the solution turns bright green until it generates brick red and the titration reaches the endpoint. The titration process is captured by a camera as shown in figure 1.

We used a camera to capture the titration process, and during the data collection process, we used an eight-megapixel autofocus auto-exposure industrial camera with a resolution of 640 × 480 pixels. The experiment was conducted in an indoor environment, where the solution to be titrated was placed in a sealed device to ensure relatively stable lighting for image acquisition. Then, an LED backlight light guide plate with adjustable brightness and chromaticity was installed at the bottom of the constructed sealed environment. During image acquisition, the brightness of the camera parameters is set to −15, contrast is set to 50, colour tone is set to 0, saturation is set to 64 and white balance is set to 4940. Use a camera to capture the titration process, then select samples of different weights and follow the same steps to collect data during the titration process. In order to ensure the accuracy and reliability of the data, the experiment was repeated many times under different lighting conditions. The collected data undergoes preprocessing, including noise removal, normalization and other steps, to ensure that the input data into the model has good quality and consistency.

### 2.2. Feature extraction

**Figure 2.**
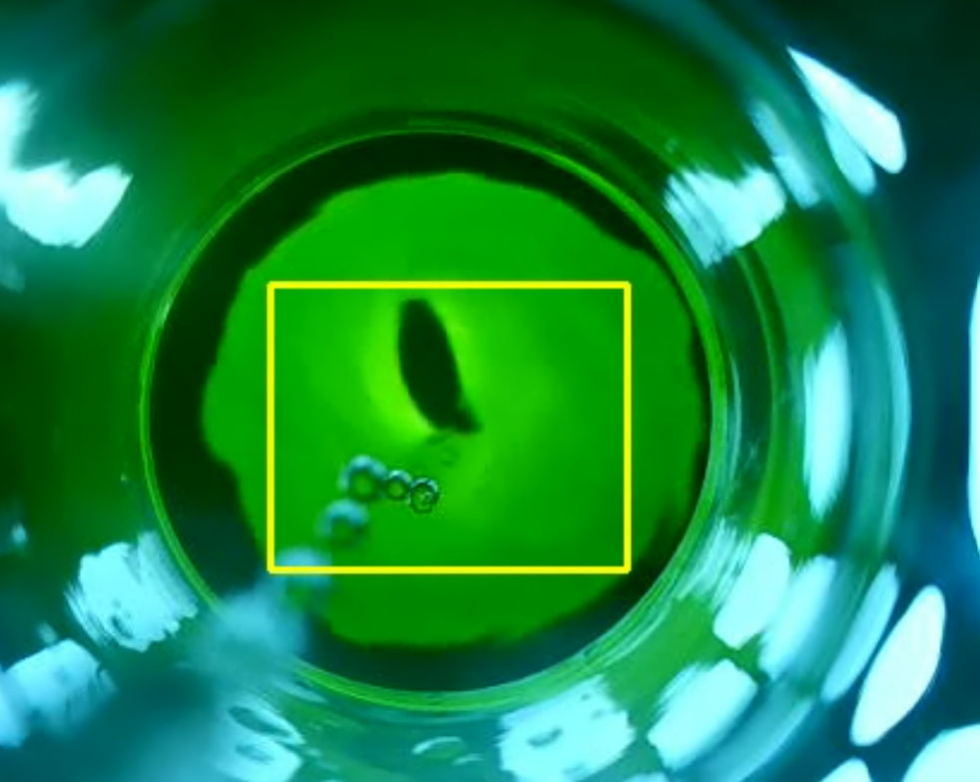
ROI area.

**Figure 3.**
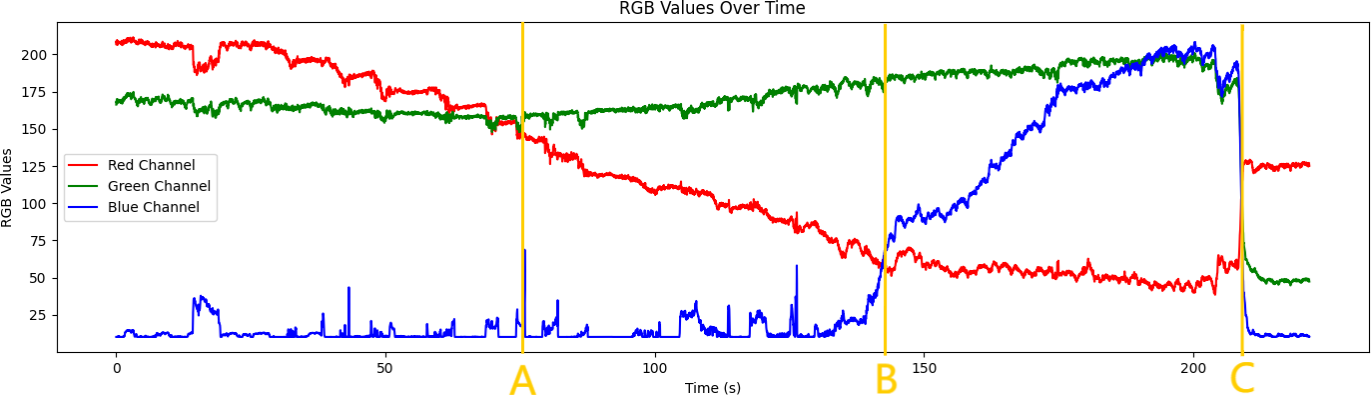
RGB value curve.

The collected video data are analysed. First, the collected video frames are processed frame by frame to determine the region of interest (ROI). The selection of the ROI area should avoid noise interference to the greatest extent. As shown in figure 2, the box area is the ROI. Subsequently, the pixels in the selected ROI are statistically analysed to calculate their average colour value in the RGB colour space. The collected RGB curve is shown in figure 3. The RGB colour space consists of three colour channels: red (R), green (G) and blue (B). The intensity value of each channel represents the brightness of the corresponding colour. In order to improve the titration efficiency, different titration speeds are used in different titration colour states. A state transition rule dictionary is defined to describe the transition conditions between different states. The states include: ‘endpoint’, ‘slow speed’, ‘medium speed’ and ‘fast speed’. The transition rule of each state is based on the relative size relationship of the current RGB value, and a default state is specified to handle unmatched situations. Then, a label mapping dictionary is created to convert the category labels (‘endpoint’, ‘slow speed ’, ‘medium speed’, ‘fast speed’) into numerical labels (0, 1, 2, 3) to facilitate the subsequent model training and evaluation.

However, considering the delay problem in the titration experiment, there may be a certain error in simply relying on RGB values to judge the titration endpoint. For this reason, the HSV colour space is further introduced, which consists of three components: hue (H), saturation (S) and value (V). Compared with RGB, the HSV colour space describes the characteristics of colour more intuitively and can better handle colour changes.

The specific steps are as follows. First, a representative image subset is selected from the collected dataset for preliminary analysis. For example, figure 3 is a representative subset. In the previous image collection process, the R, G and B values of each frame of the image are saved in a table form and marked. In figure 3, the image before point A is marked as fast speed, the image from point A to point B is marked as a medium speed, the image from point B to point C is marked as slow speed, and the image from point C and after is marked as the endpoint. The other collected data are also marked, and then these marked values are trained in the model.

Second, experimental data are collected under different time periods and lighting conditions, and the HSV values of the collected data are extracted. In multiple experiments, the HSV values of the titration endpoint are extracted, and the H-V feature histogram is drawn and used as the reference frame. Considering the colour difference of solutions of different concentrations, the similarity threshold between the reference frame and the real-time frame is set to 20%.

Finally, by combining the analysis results of the two colour spaces, RGB and HSV, the titration endpoint can be more accurately identified and confirmed, thereby improving the accuracy and reliability of the experiment.

### 2.3. Establishment of titration state model

Machine learning is a branch of artificial intelligence technology. It allows machines to automatically learn and improve performance based on data. Machine learning has been widely applied in various fields [36,37]. According to the system performance and actual needs, this article designs and establishes a titration state model, aiming to improve the system’s detection speed and efficiency of the titration process.

In order to verify the effectiveness of the model, 100 organic fertilizer samples were used for the experiment. In the specific operation, first collect video data of the sample during the titration process, and then extract colour feature values from it. Subsequently, with the help of machine learning algorithms, the four states during the titration process—slow, medium, fast and endpoint—were classified and recognized.

Given that the sample data used all have labels, this study introduces supervised learning algorithms and selects three commonly used classification methods for comparative analysis, namely decision tree model [38,39], *k*-nearest neighbours (KNN) algorithm [40] and support vector machine (SVM) model [41]. All three models can fully utilize these labelled data for training and deeply learn the mapping relationship between features and categories. After training, the model can accurately classify new and unknown data, thereby achieving precise recognition of different speed states during the titration process.

#### 2.3.1. Decision tree model

Decision trees can intuitively represent the decision-making process, similar to the way humans make decisions. Each internal node represents a test on an attribute, each branch represents a test output, and each leaf node represents a category or value. This makes the decision logic of the model very clear, easy to understand and interpret. In addition, decision trees have relatively relaxed requirements for data and do not require special preprocessing of the data. When processing data, it can also automatically handle nonlinear relationships between features. Given the nonlinear characteristics of the data involved in this article, these properties of decision trees make them an ideal choice for processing such data.

In order to evaluate the stability and generalization ability of the titration state recognition model, this article conducted multiple experiments, using different random seeds (random state) to divide the training set and test set in each experiment to ensure the reliability and repeatability of the experimental results. In the data processing stage, a colour difference threshold was set to eliminate data points with insignificant colour features, thereby reducing the impact of noise data on the model and improving the generalization ability of the model.

For unbalanced datasets, undersampling methods are used to balance the number of samples in each category to avoid classification bias caused by too many samples in a certain category. The ratio of the training set and the test set is 7 : 3 each time, that is, 70% of the data are used for training and 30% of the data are used for testing. In the model training stage, a decision tree classifier with a maximum depth of 3 is used. By limiting the maximum depth of the decision tree, the excessive growth of the tree structure can be effectively prevented, thereby avoiding the occurrence of overfitting. After the decision tree classifier is trained on the training set, predictions are made on the test set to evaluate the performance and classification accuracy of the model.

#### 2.3.2. *k*-nearest neighbours model

The principle of the KNN algorithm is concise, and as a typical lazy learning algorithm, it does not require an explicit training process. When conducting prediction work, the algorithm directly calculates and classifies based on the distance between the input sample and the training sample. This feature enables the KNN algorithm to quickly adapt to new data.

In the scenario of titration experiments, if it is necessary to quickly classify newly emerging titration states or if experimental data is continuously updated, the advantages of the KNN algorithm are highlighted. It can make decisions immediately based on new data without the need to retrain the model, greatly improving the efficiency and timeliness of experimental data processing.

The data partitioning method of the KNN model is the same as that of the decision tree model, that is, the same random seed is used to partition the training set and the test set. The difference is that after the data are partitioned, the KNN model uses the KNN classifier for model training and prediction. In this article, the parameter of the KNN model is set to *k* = 3, that is, the three nearest neighbouring points are considered to determine the classification result during classification. This setting can reduce the impact of noise on the classification result while retaining sufficient local information.

In order to further verify the stability of the KNN model, this article adopts the 10-fold cross-validation method. By dividing the dataset into 10 subsets, nine of them are used for training in turn, and the remaining one subset is used for testing, and the average performance is finally taken to verify the generalization ability of the model on different data subsets. This method can not only reduce the model’s dependence on a specific dataset, but also effectively evaluate the stability and robustness of the model.

#### 2.3.3. Support vector machine model

During the process of collecting video data and extracting colour feature values, high-dimensional feature data may be obtained. Moreover, there may be complex nonlinear relationships between the state and colour characteristics during the titration process. SVM can map low-dimensional data to high-dimensional space through kernel functions, thereby finding the optimal classification hyperplane in high-dimensional space, which can effectively handle high-dimensional data and nonlinear classification problems. And the model has strong generalization ability. During the titration process, due to possible fluctuations in experimental conditions, SVM can maintain good classification performance under different experimental conditions and accurately classify new and unknown data.

The application of the SVM model in the classification of titration states first preprocessed the data. The preprocessing stage is similar to the previous two models. The threshold of the colour feature is set to remove insignificant data points, and the undersampling method is used to process the unbalanced dataset. Subsequently, the dataset is divided into a training set and a test set, where the test set accounts for 30% of the total data. In order to ensure the stability and reliability of the experimental results, this article sets different random seeds for data partitioning experiments many times.

After each data division, the radial basis function (RBF) is used as the kernel function to construct the SVM classifier. The RBF kernel function has good performance in dealing with nonlinear classification problems and can effectively capture complex patterns in the data. The SVM classifier is trained on the training set and then predicted on the test set to evaluate the classification performance of the model. Through multiple experiments and cross-validation, the SVM model demonstrates good generalization ability and stability.

### 2.4. Design of automatic titration algorithm

By establishing a titration state model, different titration states during the titration experiment can be classified to improve the titration efficiency. However, the principle of the titration endpoint determined in this experiment is that when 1−2 drops of ammonium ferrous sulfate are added in excess, the o-phenanthroline indicator combines with Fe²^+^ to form a red complex, and the solution changes from green to brick red, at which time the titration endpoint is reached. However, since ferrous ions are not very stable and are easily oxidized to trivalent iron by air, the brick red colour at the endpoint will fade, revealing the green colour of trivalent chromium. This may be related to slow titration speed, uneven stirring, incomplete reaction, etc., so it is necessary to ensure that the colour does not return to blue within half a minute, but at this time the camera has captured the image information of the colour change moment, and it is likely that the titration endpoint has been determined and the titration is stopped. Therefore, in view of this colour instability, the time factor, that is, the colour change trend and duration, must be considered when designing the titration algorithm to ensure that the identified endpoint is accurate.

The specific steps are described in the following sections.

#### 2.4.1. Building a titration state model

By using the titration state model established by classification, the solution state during the titration process is predicted in real time. The model divides the labels of each frame into four categories:

— Label 0: titration endpoint.
— Label 1: slow speed titration.
— Label 2: medium speed titration.
— Label 3: rapid speed titration.

Based on the predicted label, the syringe pump speed is adjusted accordingly:

— Label 1 (slow speed): the syringe pump speed is set to 0.5−1 drops per second.
— Label 2 (medium speed): the syringe pump speed is set to 1−2 drops per second.
— Label 3 (fast speed): the syringe pump speed is set to 3−4 drops per second.
— Label 0 (endpoint): stop titration.

#### 2.4.2. Real-time prediction and control

The algorithm uses a camera to capture images of the solution during the titration process in real time, predicts each frame and dynamically adjusts the speed of the syringe pump. When the model predicts that a frame of image is labelled 0, it is considered that the titration endpoint has been reached and the titration process is stopped.

#### 2.4.3. Colour trend verification

**Figure 4.**
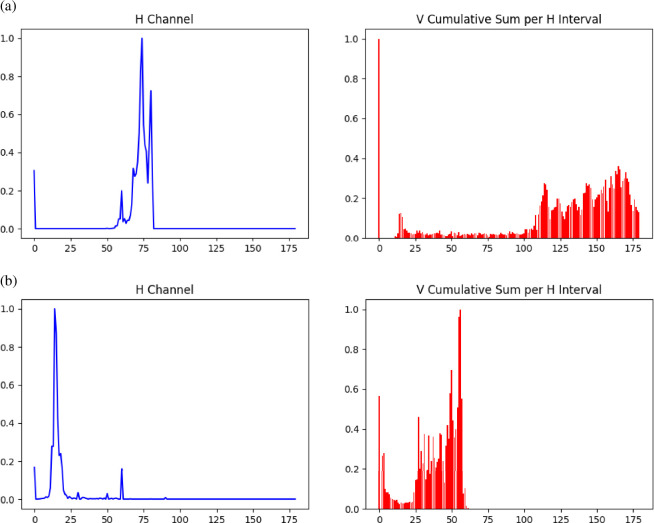
Histogram output (a) before mutation and (b) after mutation.

Since the colour at the titration endpoint may be unstable, this judgement needs to be further verified. At this time, the HSV model in the feature extraction continues to judge. After the titration state model judges to stop, the frame is recorded as *F_i_* , the H-V features of the frame and several frames before and after it are extracted, and the H-V histogram is obtained. The H-V histogram can better capture the trend of colour changes. Draw a histogram with the H channel as the horizontal axis and the cumulative sum of V in each H interval as the vertical axis. By comparing the histograms before and after the colour mutation, it is possible to more accurately judge whether the titration endpoint is stable. The histogram of two frames before and after the colour mutation is shown in figure 4.

It can be seen that there is a big difference between the HV histograms before and after the solution colour mutation. Afterwards, perform histogram similarity calculation with the reference frame *F_j_* established in the HSV model. There are usually four methods for calculating the histogram similarity, namely correlation calculation, chi-square calculation, cross calculation and Bhattacharyya distance calculation. Due to the relatively simple formula for calculating correlation. Generally, it only involves basic mathematical operations, and in actual programming implementation and calculation processes, the computational workload is relatively small and the calculation speed is fast. This is particularly important for application scenarios that require real-time processing of a large number of image frames, such as real-time video analysis, as it can significantly improve the system’s response speed and processing efficiency. In addition, the range of values for the correlation coefficient in the correlation calculation is [−1, 1], and the results are very intuitive and easy to understand. This intuitive way of representing results allows us to quickly understand the similarity between two histograms, which facilitates subsequent decision-making and analysis. Therefore, this article uses correlation calculation for histogram comparison. Correlation is a measure of the degree of linear correlation between variables. The stronger the correlation between two variables, the closer the correlation coefficient will be to ±1. The calculation formula is shown in equations (2.3) and (2.4).


(2.3) $$d\left(H_{1},H_{2}\right)=\frac{\sum_{I}\left(H_{1}(I)-\bar{H_{1}}\right)\left(H_{2}(I)-\bar{H_{2}}\right)}{\sum_{I}{\left(H_{1}(I)-\bar{H_{1}}\right)}^{2}\sum_{I}{\left(H_{2}(I)-\bar{H_{2}}\right)}^{2}},$$

(2.4) $$\bar{H_{k}}=\frac{1}{M}\sum_{{,}_{I}}H_{k}(I),$$

wherein, *H_1_* and *H_2_* represent the histogram of the real-time solution image and the histogram of the reference frame solution image, respectively, *I* represents the colour interval of the abscissa of the histogram,$\bar{H_{k}}$ represents the histogram colour mean and *M* represents the total number of colour intervals of the abscissa of the histogram, which is usually set to 255.

#### 2.4.4. Titration endpoint verification

**Figure 5.**
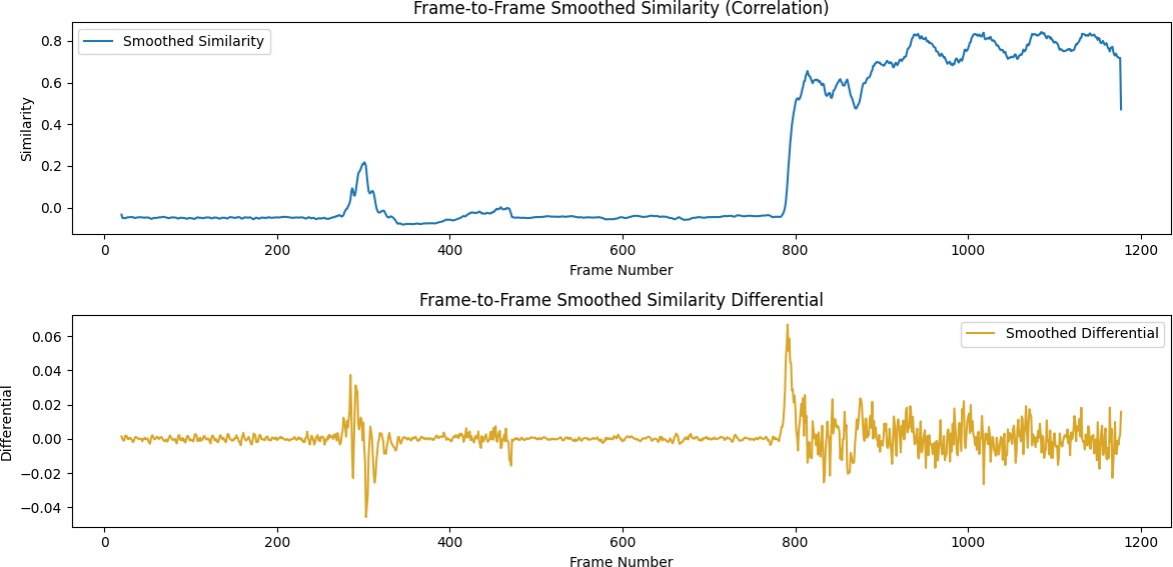
Colour histogram similarity and its differential curve.

Determine whether the similarity between *F_i_* (*n* ∈ (1…,20…,20)) and the reference frame *F_j_* reaches the set threshold. If the similarity between each frame and the reference frame *F_j_* in the next 20 frames is lower than the threshold set in the HSV model, it is considered that the titration endpoint has been reached, otherwise the titration continues. The real-time curve and differential curve of the colour histogram similarity are shown in figure 5.

As can be seen from the figure, there is a peak between 200 and 400 frames. This peak is the first colour change. At this time, the titration state model has determined it as the titration endpoint, but it fades immediately, which is caused by the incomplete reaction of the sample. At about 800 frames, another peak appears, that is, the solution turns brick red and does not fade. At this time, the chemical reaction endpoint is reached.

**Figure 6.**
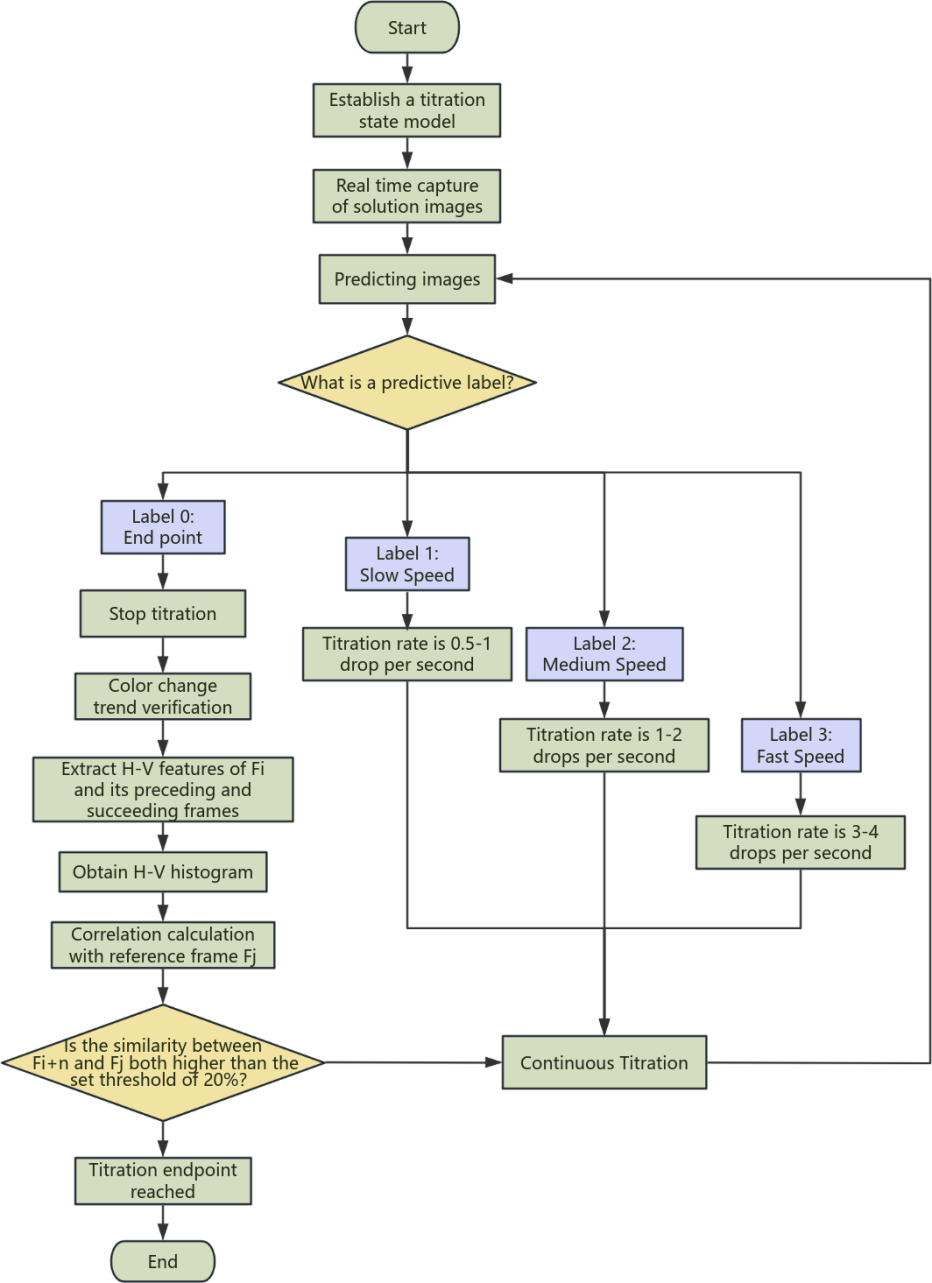
The algorithm flowchart.

The algorithm flowchart is shown in figure 6.

### 2.5. Design of automatic titration device

**Figure 7.**
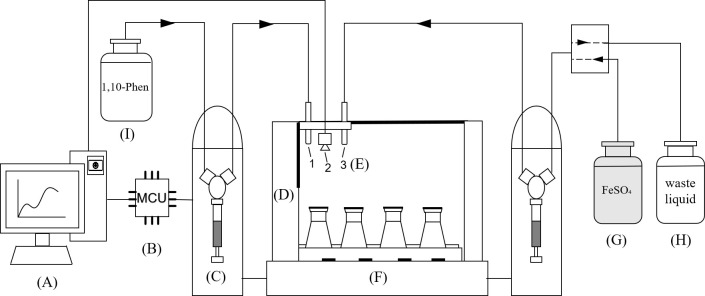
Automatic titration device. (A) Control end: including data acquisition and processing. (B) MCU: receiving control end instructions and sending them to other devices. (C) Automatic titration injection pump with 5 ml syringe and 2-port selection valve. (D) Mobile device. (E) Titration terminal, including indicator dropper (1), camera device (2) and burette (3). (F) Visual light source and magnetic stirring device. (G) Ferrous sulfate titration solution. (H) Wastewater treatment. (I) Phenanthroline indicator.

The automatic titration system is shown in figure 7. A 4 × 4 tray is placed on a platform with a visual light source and a magnetic stirring device. There are 16 bottles of samples in total. A magnetic stirring rotor is placed in each sample conical bottle. Before titration, the o-phenanthroline indicator solution and ferrous sulfate solution are filled into 5 ml syringe pumps respectively. The camera device is turned on through the titration software, and instructions are issued to the MCU at the same time. The MCU controls the visual light source and the magnetic stirring device to turn on and controls the screw-moving device to move the titration terminal to the first sample. Start titration. After the first sample is finished, the titration software processes the data and the titration terminal moves to the next sample. Repeat the whole process until the last sample is titrated. Finally, empty the ferrous sulfate solution remaining in the injection pump into the waste bottle.

## 3. Testing and analysis

### 3.1. Model performance evaluation

**Figure 8.**
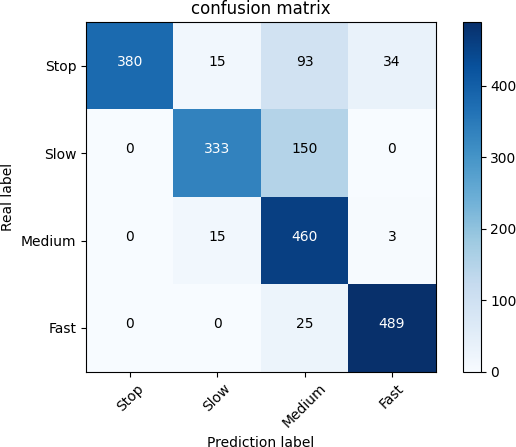
Confusion matrix.

The three classification models used in the establishment of the titration state model are evaluated for model performance and the optimal model is selected. When evaluating model performance, the confusion matrix can intuitively understand the classification performance of the model. Take the confusion matrix of the decision tree model as an example, as shown in figure 8.

**Figure 9.**
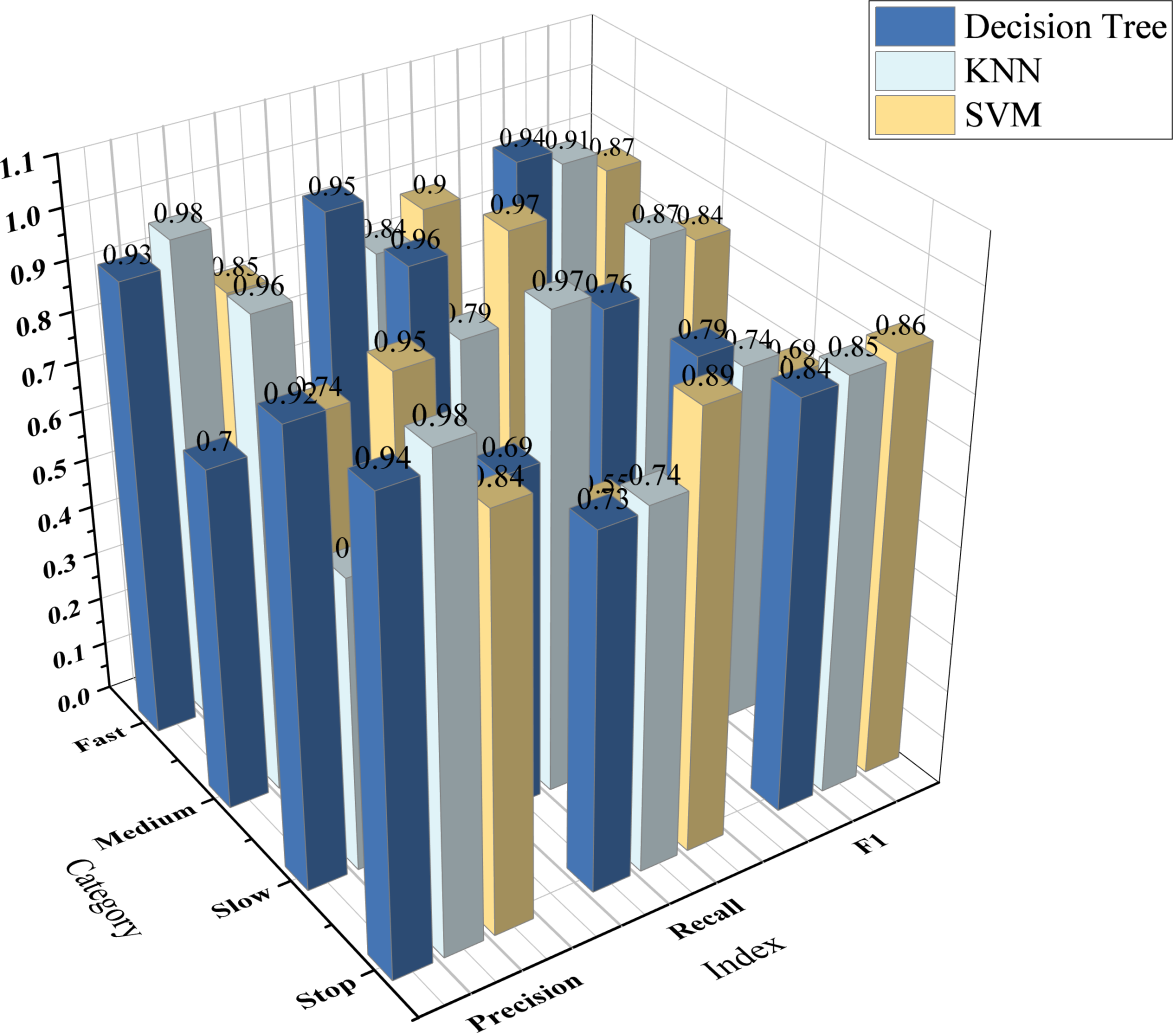
Evaluation indicators of three models in different categories.

By using the confusion matrix, performance indicators such as accuracy, precision, recall, *F1* score, *P*_micro_, *R_micro_* and *F1_micro_* can be calculated to evaluate the performance of decision tree, KNN and SVM classification models. These evaluation indicators are defined as follows:


(3.1) $$Accuracy=\frac{TP+TN}{TP+TN+FP+FN},$$

(3.2) $$Precision=\frac{TP}{\left(TP+FP\right)},$$

(3.3) $$Recall=\frac{TP}{\left(TP+FN\right)},$$

(3.4) $$F1=\text{2}\times \frac{Precision\times Recall}{\left(Precision+Recall\right)},$$

(3.5) $$P_{\text{micro }}=\frac{\sum_{i=1}^{N}TP_{i}}{\sum_{i=1}^{N}TP_{i}+\sum_{i=1}^{N}FP_{i}},$$

(3.6) $$R_{\text{micro }}=\frac{\sum_{i=1}^{N}TP_{i}}{\sum_{i=1}^{N}TP_{i}+\sum_{i=1}^{N}FN_{i}},$$

(3.7) $$F1_{\text{micro}}=\frac{2\times P_{\text{micro}}\times R_{\text{micro}}}{P_{\text{micro}}+R_{\text{micro}}},$$

(*i*, *j*) in the confusion matrix represents the number of samples whose actual class is *i* that are predicted to be class *j*. Among them, TP represents the number of samples correctly predicted to be class *i* by the model for each class *i* . TN represents the number of samples correctly predicted to be other classes (not *i*) by the model for each class *i*. FP represents the number of samples incorrectly predicted to be class *i* by the model for each class *i* (actually other classes). FN represents the number of samples incorrectly predicted to be other classes (not *i*) by the model for each class *i* (actually class *i*). *N* represents the sample class. For the four classes, the classification evaluation indicators of each model are shown in figure 9.

As can be seen from figure 9, the three models of decision tree, KNN and SVM have different precision, recall and F1 scores for the four categories of ‘stop’, ‘slow’, ‘medium’ and ‘fast’. Among them, the overall performance of the decision tree model is good, all of which are greater than 70%. The precision of the KNN model is relatively low, and the precision for medium speed category recognition is as low as 60%, but it has a higher recall rate. The overall performance of the SVM model is lower than that of the other two models, The recall rate for the slow category is as low as 55% and the F1 score is also below 70%.

**Figure 10.**
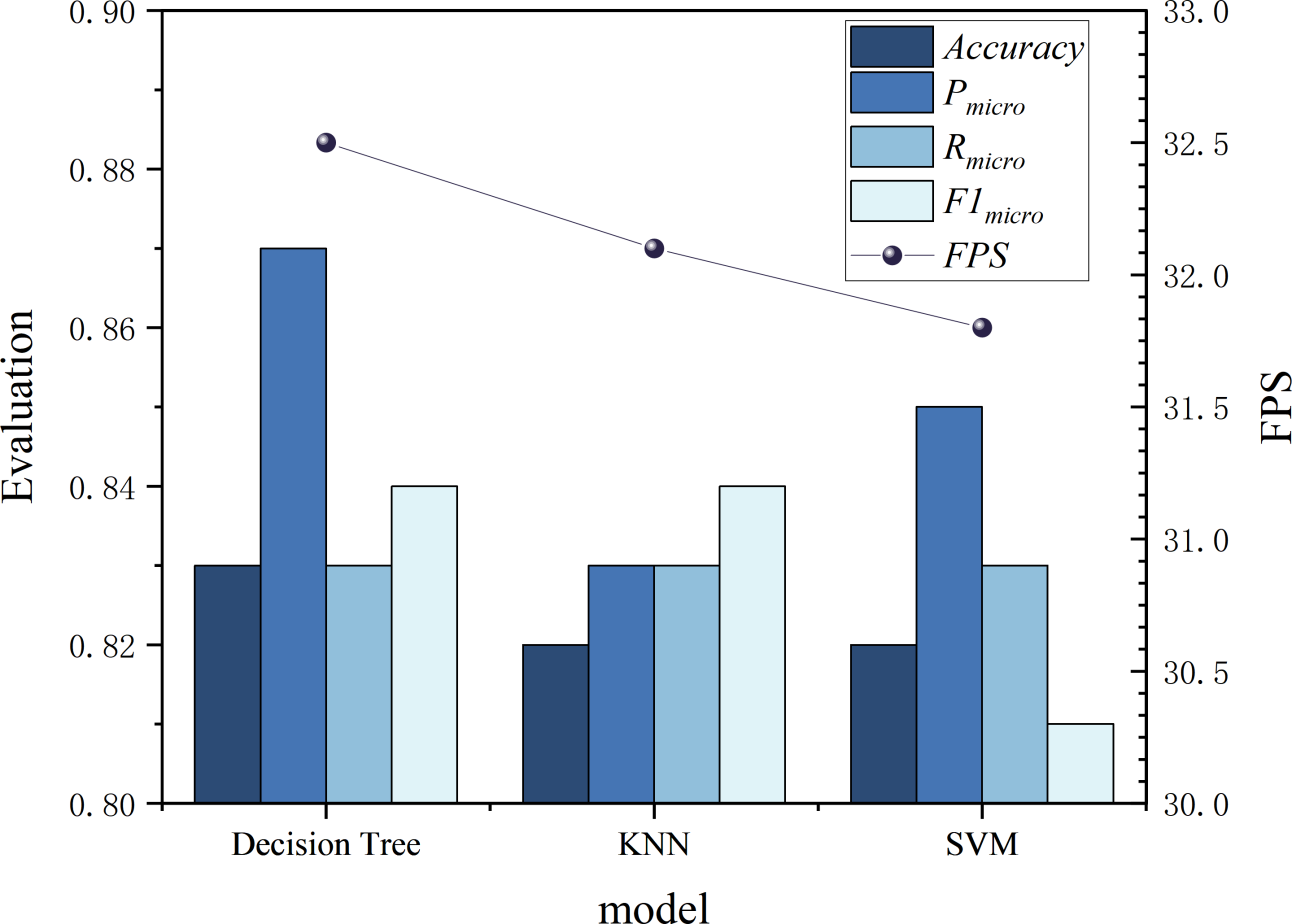
Evaluation indicators of each model.

In addition, since the data of this experiment is video data, when applying the model, each frame of the video data needs to be extracted and converted into a numerical type, so the processing speed of the model needs to be fast enough. FPS (the number of image frames that the model can process per second) is an important and intuitive indicator that reflects the processing speed of the model. The overall evaluation indicators of each model are shown in figure 10.

As can be seen from figure 10, the various indicators of the decision tree perform better than the other two models, with an accuracy rate of 83.22%. At the same time, the FPS represents the model processing speed, which is also better than other models and can reach 32.5.

In summary, the establishment of the titration state model in this system selects a decision tree model with higher accuracy and faster image processing speed per frame.

### 3.2. Performance test of automatic titration system

In order to verify that the automatic titration algorithm designed in this article is accurate in judging the titration endpoint and that the established titration state model is more efficient than constant-rate titration and manual titration, this article tests it from three aspects: response time, accuracy and stability.

Before conducting the experiment, it is necessary to make sufficient preparations. Prepare organic fertilizer samples of different qualities and prepare the solution according to the potassium dichromate volumetric method mentioned in §2.1. After the solution preparation is completed, add o-phenanthroline indicator dropwise to each titration solution.

Then, the test is carried out from three key aspects. The first is the response time test. Multiple groups of experiments are carried out using the automatic titration algorithm, constant-rate titration and manual titration. During the experiment, the time spent from the start of titration to the endpoint is accurately recorded to evaluate the response efficiency of different titration methods.

The second is the accuracy test. Multiple groups of experiments are also carried out. The experimental results of the automatic titration algorithm and the manual titration method are carefully compared, and the difference between the two is calculated. Based on this, the accuracy of the automatic titration algorithm for identifying the titration endpoint is evaluated.

Finally, the stability test is carried out. The automatic titration algorithm is used multiple times to carry out experiments under different time periods and diverse environmental conditions. The volume of titrant consumed in each experiment is recorded, and these data are deeply analysed to calculate the relative standard deviation. Through this series of tests and analyses, the performance of the automatic titration algorithm is comprehensively evaluated.

#### 3.2.1. Response time test

**Figure 11.**
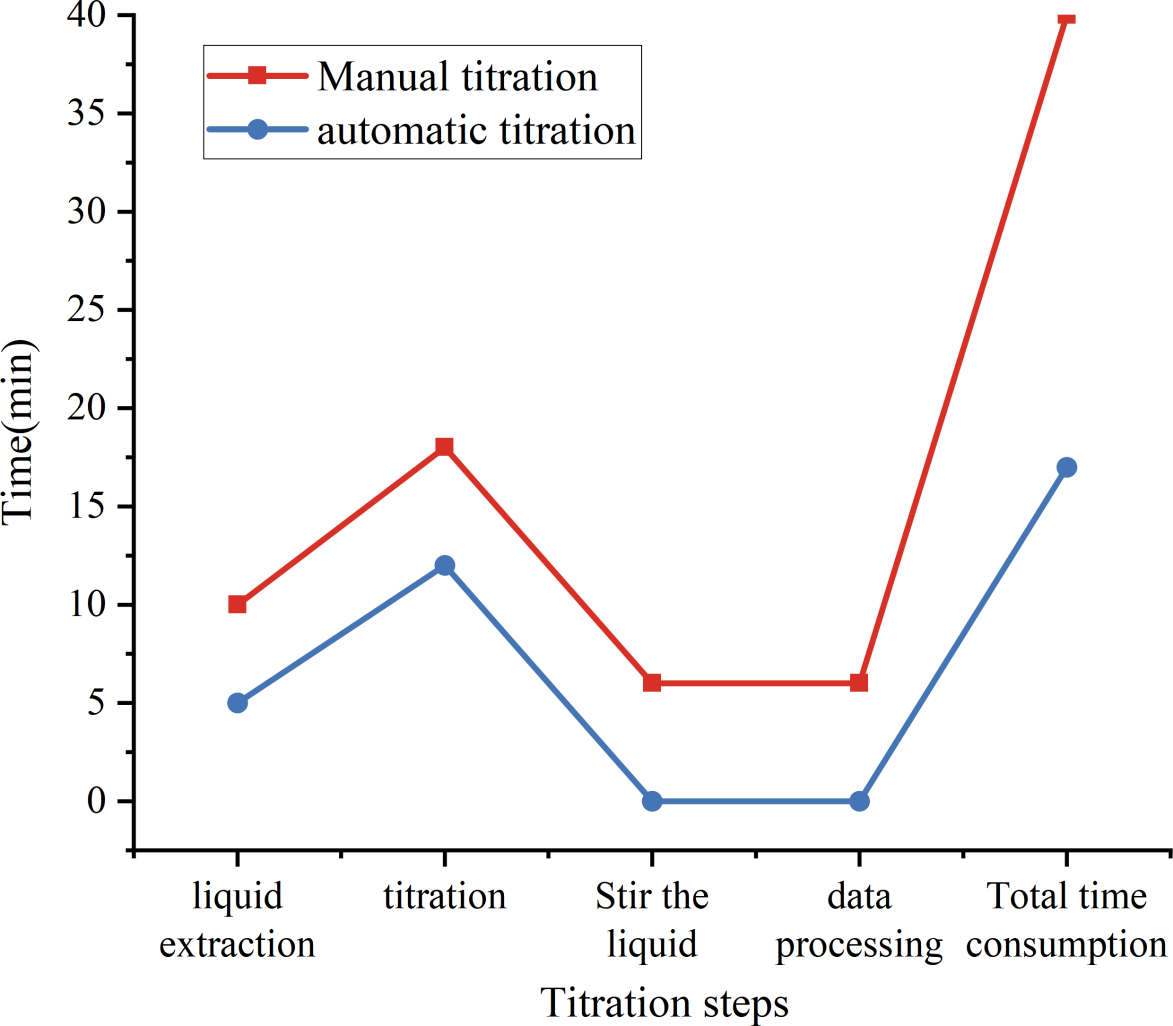
Time comparison with manual titration test. This is the total time consumed for the titration of six samples. The stirring and data processing of the instrument are carried out simultaneously during the titration and liquid extraction process, so it is 0.

In order to evaluate the time required for the algorithm in this article to complete a titration and ensure that it meets the experimental requirements, it is compared with constant-rate titration and manual titration to verify whether it can complete the titration experiment faster. Here, the titration of six samples is taken as an example, and traditional manual titration and constant-rate device titration are performed at the same time. The total time is calculated by comparing liquid extraction, titration, stirring and data processing. The comparison with the time consumption of the traditional manual titration test is shown in figure 11.

It can be clearly seen from figure 11 that the traditional manual titration process involves multiple time-consuming steps, including dripping, titration, stirring and data processing, etc. These steps usually need to be performed in sequence and cannot be performed in parallel. Therefore, the entire titration process is time-consuming and inefficient.

In contrast, this system does not require separate time for titration data processing and stirring. Titration and stirring operations can be performed simultaneously, without waiting for the stirring to be completed before titration as in manual operation. This design not only reduces the operation time, but also ensures uniform stirring during the titration process, thereby improving the accuracy of the titration. In addition, data processing does not require additional calculations. When the system starts to titrate the next sample, the data processing of the previous sample has been completed, thus achieving zero waiting time for data processing.

**Figure 12.**
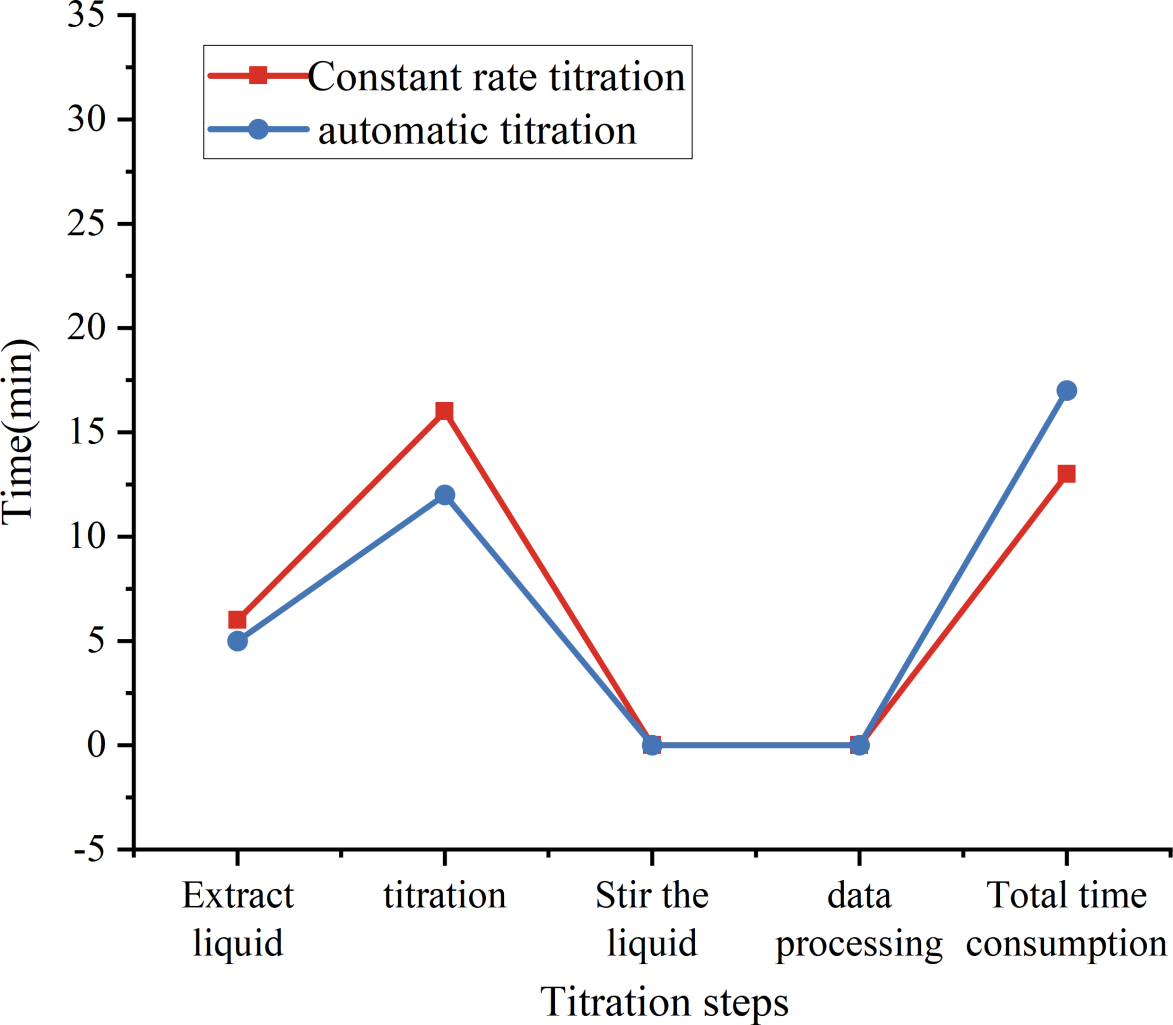
Comparison of time consumption with a constant-rate titration test.

In addition, the algorithm in this article is compared with the constant-rate titration system. Constant-rate titration is titration at a constant rate all the time. No titration state model is established. The endpoint is identified only by comparing with the established HSV model. The time consumption comparison with the constant-rate titration test is shown in figure 12.

From figure 12 that the two methods have obvious differences in the titration speed. The algorithm in this article can automatically adjust the titration speed according to the current titration state by establishing a titration state model. For example, in the early stage of titration, the system will use a faster titration speed to shorten the overall time; when approaching the endpoint, the system will automatically slow down the titration speed to ensure accurate capture of the titration endpoint. This intelligent speed control further improves the efficiency and accuracy of titration.

#### 3.2.2. Accuracy test

**Figure 13.**
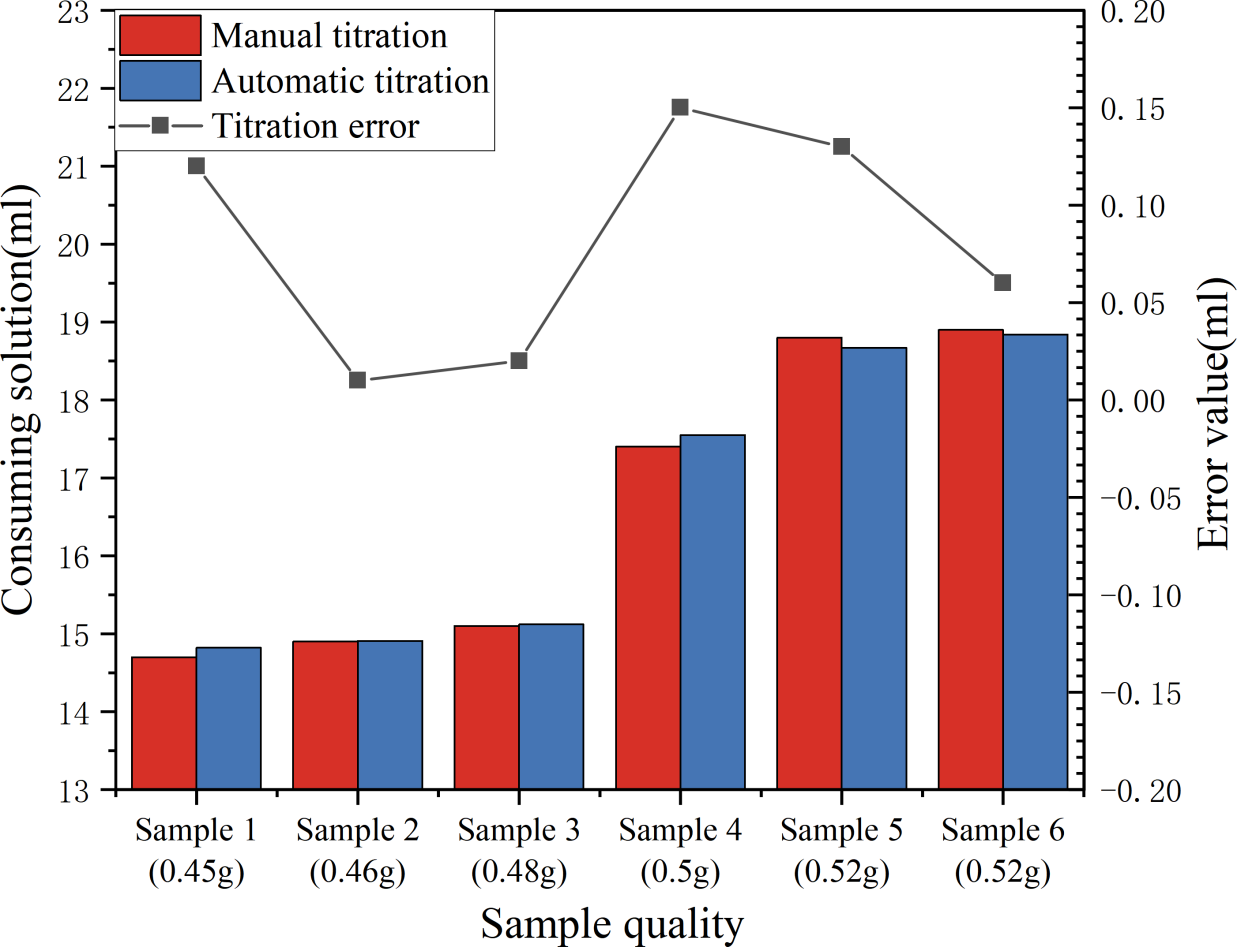
Accuracy comparison. The sample mass refers to the mass of organic fertilizer. Both the manual result and the instrument result refer to the volume of ferrous sulfate consumed in the titration solution. The content of organic matter needs to be further calculated by chemical formula and will not be calculated here.

Through multiple experiments, the accuracy of the automatic titration algorithm in judging the titration endpoint is verified to ensure the consistency of its results with standard methods (such as manual titration). This article takes the titration of six samples as an example, and conducts traditional manual titration at the same time. For the same sample, the volume of ferrous sulfate consumed is used to verify whether the judgement of the titration endpoint is accurate. The accurate comparison is shown in figure 13. On this basis, we also calculated the corresponding sample mean, standard deviation and degrees of freedom to calculate the confidence interval.

As can be seen from figure 13, the volume deviation between automatic titration and manual titration is small, and the deviation of the sample is within 0.2 ml. The relative error is also small, and the relative error of most samples is within 1%. These intuitive data preliminarily show that the automatic titration algorithm has a high accuracy in judging the titration endpoint.

In order to further verify whether there is a significant difference between the automatic titration algorithm and the manual titration method from a statistical point of view, we applied a paired *t*‐test at a 95% confidence level. The paired *t*‐test is a commonly used statistical method that is suitable for comparing the results of two related measurement methods. After calculation, the mean is −0.0167, the standard deviation is 0.248, the *t*-value is 0.17 and the degree of freedom *d_f_* = 5 (the degree of freedom refers to the number of data that can be freely taken in statistical calculations, where the degree of freedom is the number of samples minus 1). By consulting the *t* distribution table or using statistical software for a two-tailed test, the *p* value is 0.87, and the significance level we set is *α* = 0.05. In statistics, when the *p* value is greater than the significance level *α*, we believe that there is no statistically significant difference between the two methods. That is to say, at a confidence level of 95%, the results of the automatic titration algorithm and the manual titration method in determining the titration endpoint are consistent. Therefore, the automatic titration algorithm used in this article has high recognition accuracy, and its titration endpoint determination results are highly consistent with the manual titration method, which can basically meet the expected performance indicators.

#### 3.2.3. Stability test

In order to further verify the stability of the automatic titration algorithm in practical applications, this article designed a series of stability tests, focusing on the stability of the automatic titration algorithm in judging the titration endpoint under different experimental conditions.

First, we tested the time stability by testing it at different time periods on the same day. The test results are shown in table 1. Then, we tested it in different laboratory environments to verify the environmental stability. The test results are shown in table 2.

**Table 1.** Time stability test results. Absolute difference = (single measurement value − average value), referring to NY/T 525-2021 standard; the absolute difference of parallel samples should be ≤ 1.0%.

sample number	time period	titration volume (ml)	organic matter content (%)	average value of organic matter (%)	absolute difference (%)
S1	morning	18.24	45.3	45.3	0.0
	afternoon	18.37	45.1		0.2
	night	18.12	45.6		0.3
S2	morning	18.68	46.7	46.6	0.1
	afternoon	18.53	46.5		0.1
	night	18.87	47.0		0.4
S3	morning	17.93	42.6	42.6	0.0
	afternoon	17.82	43.2		0.6
	night	18.07	41.9		0.7
S4	morning	18.38	48.1	48.0	0.1
	afternoon	18.32	47.8		0.2
	night	18.51	48.4		0.4

**Table 2.** Environmental stability test results. The samples here are from the same batch of organic fertilizer, divided into six portions. Two parallel samples (numbered T20-1/T20-2, T25-1/T25-2, T30-1/T30-2) are taken at each temperature (20°C, 25°C and 30°C). The samples are titrated by adjusting the indoor temperature.

temperature (°C)	sample number	titration volume (ml)	organic matter content (%)	average value of organic matter (%)	absolute difference (%)
low temperature (20°C)	T20-1	18.34	46.3	46.7	0.4
	T20-2	18.62	47.1		0.4
normal temperature (25°C)	T25-1	17.73	47.8	47.4	0.4
	T25-2	17.89	47.0		0.6
high temperature (30°C)	T30-1	17.96	46.5	46.6	0.1
	T30-2	18.17	46.7		0.1

It can be seen from tables 1 and 2 that the automatic titration method in this article shows good stability at different time periods within a day, and changes in ambient temperature have no significant effect on the titration results. In addition, the absolute differences also comply with the NY/T 525-2021 standard, further confirming the stability of the algorithm proposed in this article.

Through the above tests, it is verified that the automatic titration algorithm designed in this article not only has advantages in the accuracy of titration endpoint judgement, but also has advantages in response time over traditional constant-rate titration and manual titration methods. In addition, under different time and environment, it has no effect on the automatic titration in this article, and the stability performance meets the requirements.

## 4. Conclusion

This article introduces machine learning technology and proposes an automatic titration algorithm for organic matter content detection based on colour features, aiming to solve the problems of cumbersome operation, environmental pollution and insufficient detection accuracy in traditional manual titration methods. A titration experiment state recognition model is established through a decision tree classifier with an accuracy rate of 83.22%. This article realizes the intelligent classification of titration speed and designs an efficient titration algorithm, which effectively improves the judgement accuracy of the titration endpoint.

The experimental results show that the algorithm not only significantly improves the detection efficiency, but also reduces the errors caused by manual operation. Its accuracy can meet the requirements and has high application value. In the future, with the further development of technology, the algorithm is expected to be more widely used in agriculture, environmental protection, scientific research and other fields, promoting the process of chemical analysis automation. In addition, the research in this article also provides new ideas for the automation of other chemical analysis methods, showing the broad prospects of machine learning technology in the field of chemical detection.

However, current methods and models still have certain limitations. First, although physical means are used to block light and an LED light board is installed in the light shield as a light source, there may be some problems with the luminous stability of the LED light board itself. As the use time increases, the LED lamp beads may experience light decay, resulting in changes in light intensity and colour temperature, which in turn affects the accuracy of extracting the colour characteristics of the solution, affecting the performance of the titration experiment state recognition model. Second, although the decision tree classifier has an accuracy rate of 83.22%, it has limited ability to distinguish different chemical substances or complex chemical systems with similar colour change characteristics. In practical applications, misjudgements may occur, affecting the intelligent classification of titration speed and the accurate judgement of titration endpoint. Furthermore, the current algorithm data samples mainly focus on titration experiments related to organic matter content detection. For other types of chemical titration experiments, the lack of data may lead to insufficient generalization of the model and make it difficult to directly apply it to different scenarios.

In response to these limitations, the following improvement measures can be taken in the future. For the stability of LED light panels, the light panels can be calibrated and maintained regularly, and light sensors can be installed to monitor the changes in light intensity and colour temperature in real time, and the output parameters of the light panels can be dynamically adjusted through feedback mechanisms to ensure the consistency of lighting conditions. In terms of improving the performance of decision tree classifiers, ensemble learning methods, such as random forest algorithms, can be combined to improve the model’s recognition and anti-interference capabilities for complex chemical systems by building multiple decision trees and integrating their results. In order to enhance the versatility of the algorithm, more data from different types of chemical titration experiments should be collected, and existing models should be retrained and optimized. Transfer learning technology can be used to transfer the features and patterns learned in organic matter content detection experiments to other chemical experiments, reducing the cost of data collection and model training, thereby expanding the scope of application of the algorithm. Future research directions can also include further optimizing equipment performance, improving titration speed and accuracy, expanding the scope of application to cover more chemical experimental needs, and exploring application potential in other fields. Future research directions may include further optimizing device performance, improving titration speed and accuracy, expanding the scope of application to cover more chemical experimental needs and exploring application potential in other fields.

## Data Availability

All relevant data are within the manuscript and the electronic supplementary material [42].
